# Fucose modifies short chain fatty acid and H_2_S formation through alterations of microbial cross-feeding activities

**DOI:** 10.1093/femsec/fiad107

**Published:** 2023-09-30

**Authors:** Karina Høgsgaard, Natalia P Vidal, Angeliki Marietou, Oliver Gam Fiehn, Qing Li, Julia Bechtner, Jacopo Catalano, Mario M Martinez, Clarissa Schwab

**Affiliations:** Functional Microbe Technology Group, Department of Biological and Chemical Engineering, Aarhus University, Gustav Wieds Vej 10, 8000 Aarhus, Denmark; Center for Innovative Food (CiFOOD), Department of Food Science, Aarhus University, AgroFood Park 48, 9200 Aarhus N, Denmark; Aarhus Institute of Advanced Studies, Aarhus University, Høegh-Guldbergs Gade 6B, 8000 Aarhus, Denmark; Functional Microbe Technology Group, Department of Biological and Chemical Engineering, Aarhus University, Gustav Wieds Vej 10, 8000 Aarhus, Denmark; Functional Microbe Technology Group, Department of Biological and Chemical Engineering, Aarhus University, Gustav Wieds Vej 10, 8000 Aarhus, Denmark; Functional Microbe Technology Group, Department of Biological and Chemical Engineering, Aarhus University, Gustav Wieds Vej 10, 8000 Aarhus, Denmark; Center for Innovative Food (CiFOOD), Department of Food Science, Aarhus University, AgroFood Park 48, 9200 Aarhus N, Denmark; Membrane Engineering Group, Department of Biological and Chemical Engineering, Aarhus University, Åbogade 40. 8200 Aarhus N, Denmark; Center for Innovative Food (CiFOOD), Department of Food Science, Aarhus University, AgroFood Park 48, 9200 Aarhus N, Denmark; Functional Microbe Technology Group, Department of Biological and Chemical Engineering, Aarhus University, Gustav Wieds Vej 10, 8000 Aarhus, Denmark

**Keywords:** cross-feeding, fermentation, fucose, gut microbiota

## Abstract

Algae are a rich but unexplored source of fibers with the potential to contribute to the next generation of prebiotics. The sulfated brown algae polysaccharide, fucoidan, is mainly composed of the deoxy-hexose L-fucose, which can be metabolized to 1,2-propanediol (1,2-PD) or lactate by gut microbes as precursors of propionate and butyrate. It was the aim of this study to investigate the impact of fucoidan on the fermentation capacity of the fecal microbiota and to compare to fucose. In batch fermentations of fecal microbiota collected from 17 donor samples, fucose promoted the production of propionate while no consistent effect was observed for commercial fucoidan and *Fucus vesiculosus* extract prepared in this study containing laminarin and fucoidan. H_2_S production was detected under all tested conditions, and levels were significantly lower in the presence of fucose in a dose-dependent manner. The addition of high fucose levels led to higher relative abundance of microbial 1,2-PD and lactate cross-feeders. Our results highlight that fucose and not fucoidan addition impacted fermentation capacity and increased the proportions of propionate and butyrate, which allows for precise modulation of intestinal microbiota activity.

## Teaser

Fucose modifies fermentation activity and gas formation through the provision of pathway intermediates.

## Introduction

In comparison to societies from the Amazon, Malawi, or Papua New Guinea, gut microbiomes from Western cultures possess a lower abundance of fibre-fermenting bacterial species, which is associated with shifts in fermentation activity and the production of detrimental metabolites (Yatsunenko et al. 2012, Martinez et al. 2015). The observed link between health, diet, dietary fibre, and gut microbiome has led to a renaissance of prebiotics to address a gap in nutrition caused by contemporary dietary habits. The International Scientific Association for Probiotics and Prebiotics (ISAPP) defines a prebiotic as “a substrate that is selectively utilized by host microorganisms conferring a health benefit” (Gibson et al. 2017). Most dietary prebiotics are non-digestible, soluble carbohydrates that reach the colon without being hydrolyzed by pancreatic and intestinal enzymes (Gibson et al. 2017). The colon has the highest microbial cell density in the gastrointestinal tract (up to 10^11^ cells per gram of gut content, Derrien and Van Hylckama Vlieg 2015, Vandeputte et al. 2017) with *Bacillota* (former *Firmicutes*), *Bacteroidota* (former *Bacteroidetes*), *Pseudomonadota* (former *Proteabacteria*), *Actinomycetota* (former *Actinobacteria*) and *Verrucomicrobiota* (former *Verrucomicrobia*) as the most abundant phyla. Colonic microbes use the non-digestible carbohydrates as substrate for fermentation processes.

**Figure 1.**
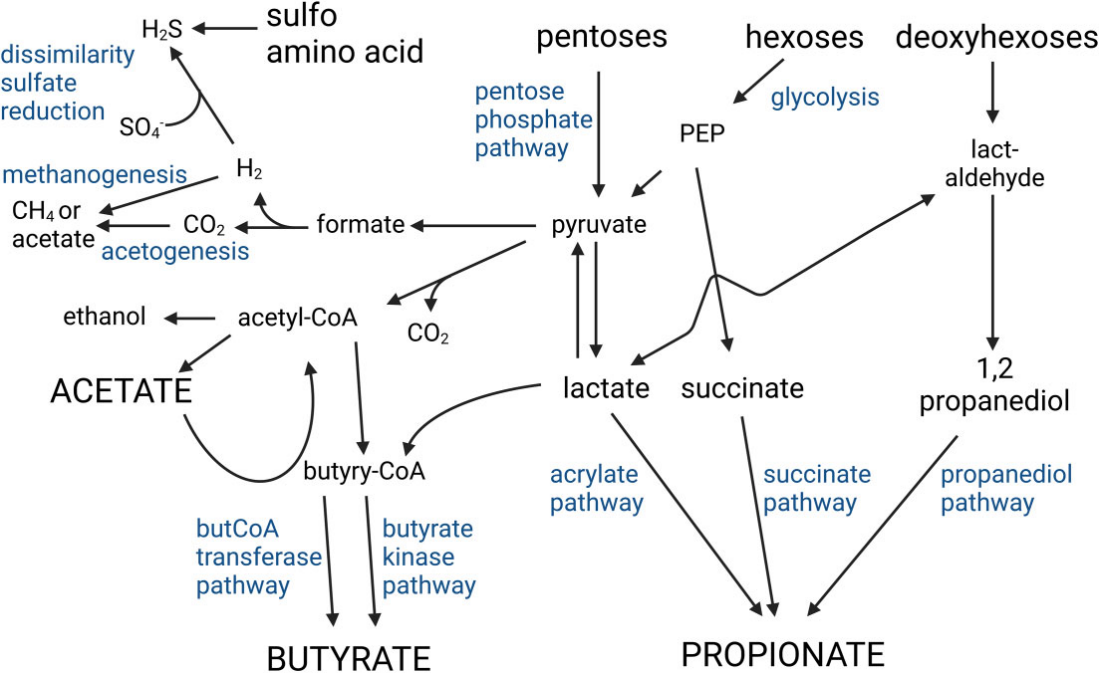
Scheme depicting major fermentative pathways of gut microbes. Microbial cross-feeding possibilities in the presence of hexoses, pentoses, deoxyhexoses, and amino acids. Figure was prepared with BioRender.

The utilization of carbohydrates by intestinal microbial encompasses polymer degradation and fermentation as primary processes (Fig. 1). Non-digestible carbohydrates are enzymatically hydrolysed to hexoses (e.g. glucose, galactose, fructose), deoxy-hexoses (e.g. rhamnose, fucose) and pentoses (e.g. arabinose, xylose) (Flint et al. 2012). Hexoses and pentoses are oxidized to pyruvate, which acts as the main precursor for short chain fatty acid (SCFA) formation; additionally SCFA are formed through microbial metabolite cross-feeding (Fig. 1). Most gut microbes produce acetate as a fermentation product (Louis and Flint 2017). Butyrate is produced via the butyrate kinase pathway or the butyryl CoA: acetyl CoA transferase pathway (Fig. 1). Some microbes form butyrate from lactate and acetate (Louis and Flint 2017). Propionate is produced from the fermentation intermediates succinate (succinate pathway), lactate (acrylate pathway) or 1,2-propanediol (1,2PD, 1,2-PD pathway) (Louis and Flint 2017). Fermentation metabolites 1,2-PD, lactate, succinate and formate do typically not reach a high concentration in fecal samples from healthy adults as they can be further metabolized by cross-feeding (Fig. 1). Propionate and butyrate and have been linked to beneficial health outcomes (Gibson et al. 2017).

The probably most studied prebiotics are inulin, fructo- and galactooligosaccharides (FOS and GOS) together with resistant starch. These fibres are isolated from terrestrial plants or produced biotechnologically, and are characterized by simple composition and repetitive structures. FOS, GOS and resistant starch are composed of few monosaccharides, namely fructose, galactose, and glucose, which are connected by a limited number of glycosidic linkage types. Current concepts related to precise microbiome engineering suggest the use of structurally and compositionally different carbohydrate polymers as novel prebiotics (Deehan et al. 2020, 2022). The marine ecosystem, for example algae, is a rich, underexplored natural resource of fibres that differ in composition and structure from their terrestrial counterparts (Gotteland et al. 2020). Brown algae contain the polysaccharides alginate (mannuronic and guluronic monomers), laminarin (β-linked glucose monomers) and fucoidan, which is a unique natural polymer mainly composed of fucose in L-configuration and high degree of sulfation (SO_4_^2−^, Gotteland et al. 2020). Fucose can be metabolized to 1,2-PD as a precursor of propionate (Bunesova et al. 2016, Schwab et al. 2017), and to lactate (Becerra et al. 2015).

Glycosulfatases catalyse the release of sulfate (Corfield et al. 1992), which can be reduced stepwise to sulfite (SO_3_^2−^) and hydrogen sulfide (H_2_S) by sulfate reducing bacteria (SRB) like *Desulfovibrionaceae*, which harbour the enzyme dissimilatory sulfite reductase (DSR) enzyme (Christophersen et al. 2011).

While the potential of fucoidan to act as a source of propionate can be beneficial within the gut ecosystem, there is the risk of excessive formation of H_2_S from sulfate. High levels of colonic H_2_S have been linked to the inhibition of the mitochondrial respiratory chain, lower mucosal integrity through genotoxicity reduction of mucosal disulfide bonds and inhibition of colonocyte butyrate oxidation through cytochrome-c inhibition (Blachier et al. 2021). Therefore, it is of high importance to determine whether microbial gut fermentation of fucoidan promotes H_2_S production. The overall objective of this study was to systematically investigate the fermentation of fucoidan polysaccharides from marine biomass by fecal microbiota to establish the potential of fucoidan as next generation prebiotic. Two fucoidan polysaccharides were examined in fecal microbiota batch fermentations and compared to L-fucose: a commercial fucoidan and an extract from *Fucus vesiculosus* obtained in this study.

## Materials and methods

### Experimental set-up

**Figure 2.**
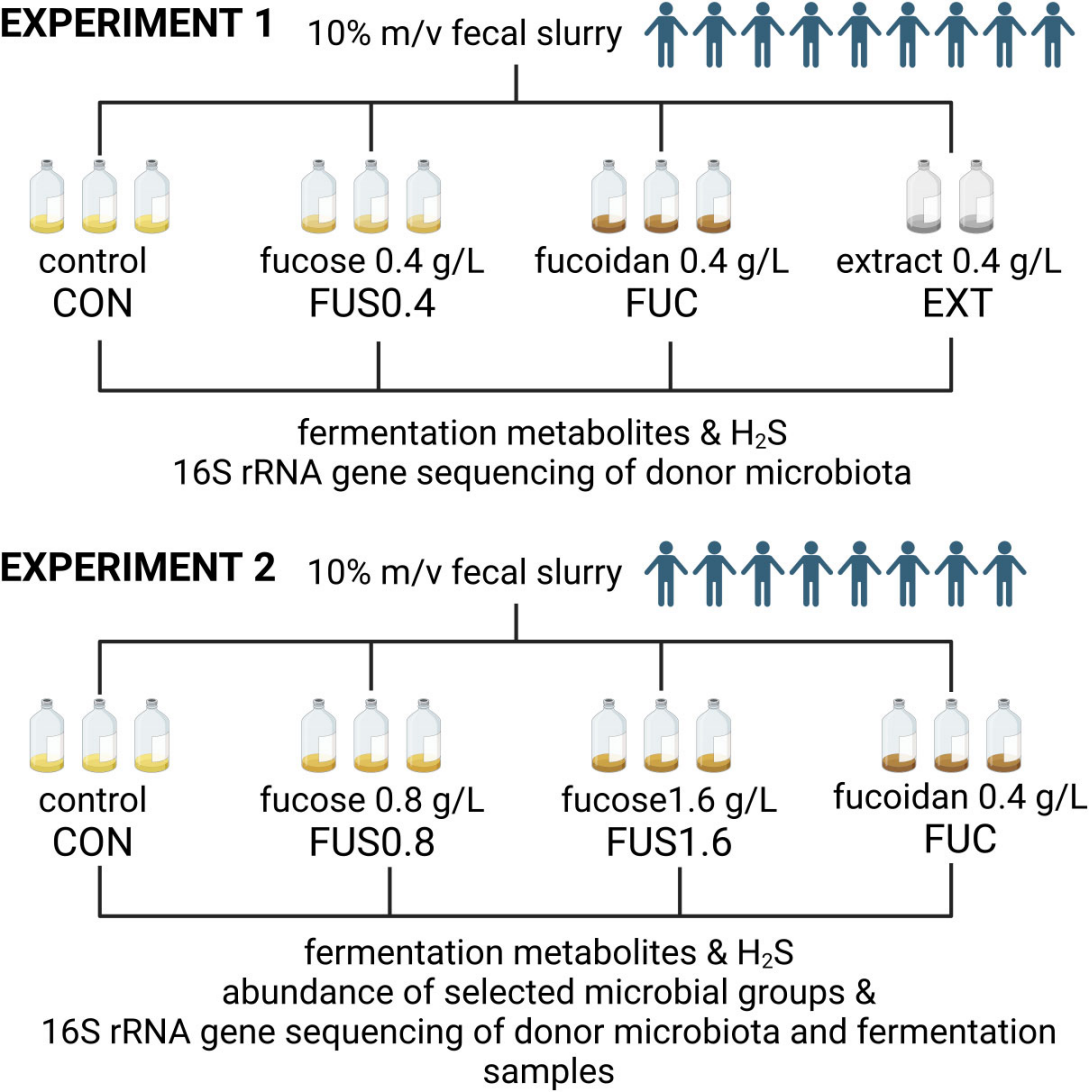
Experimental set-up of batch fermentations and analysis. Donor, treatments and major analysis conducted in experiment 1 and experiment 2. Figure was prepared with BioRender.

We first extracted and characterized the major polysaccharides from *F. vesiculosus* (Suppl. Fig. S1). Next, two *in vitro batch* fermentation experiments were conducted that used MacFarlane as base medium (Fig. 2). In experiment 1, we investigated the impact of the addition of 0.4 g L ^−1^ fucose (FUS0.4) or commercially available fucoidan (FUC, Merck), or our extract from *Fucus vesiculosus* (EXT). Batch fermentations inoculated with slurries prepared from nine donors were compared to controls (CON) using basic MacFarlane medium. In experiment 2, fucose was added at 0.8 (FUS0.8) and 1.6 (FUS1.6) g L^−1^, whereas commercial fucoidan was supplied at 0.4 g L^−1^ (FUC) (Fig. 2). We determined the concentrations of SCFA at 0, 24, and 48 h of incubation and analysed H_2_S levels at 0 and 48 h. Microbial composition and the abundance of selected bacterial groups was determined with 16S rRNA gene sequencing and qPCR using fecal samples and biomass from CON and FUS1.6 fermentation collected in experiment 2.

### Extraction of fucoidan from *F. vesiculosus*

*F. vesiculosus* was chosen for extraction as this species was the source of the commercial fucoidan (Merck, Denmark). Fresh *F. vesiculosus* was collected in September 2021 at the Aarhus Bay coast in the proximity of Aarhus (Denmark). The seaweed was washed with distilled water to remove sand and impurities and lyophilized (Christ, Gamma, 1–16, LSC). The dried seaweed was ground into powder (0.5 mm mesh, Retsch, ZM200) and was vacuum packed and stored at −20 °C until further processing. Seaweed polysaccharides were extracted as specified by Ptak et al. (2019) with modifications (Suppl. Fig. S1). Briefly, seaweed powder (60 g) was resuspended in 600 ml EtOH (95% v/v) and was stirred for 4 h at room temperature. The suspension was centrifuged, 4600 r/m, 30 min, 20°C (Heraeus, MULTIFUGE 3 S-R). The pellet was washed with 100 ml acetone 99.5 (v/v%), centrifuged and dried in an oven (Memmert, UF55) at 35°C overnight. The dried material was vacuum packed and stored at −20°C before extraction. Before extraction, dried pellets (1.5 g) were mixed with 45 ml 0.1 M HCl, and microwave extracted (Anton Paar Multiwave 3000) with a power-gradient: ramp from 0 to 300 W for 5 min, hold at 300 W for 15 min (fan 1), followed by 0 W for 5 min (fan 3). The maximum temperature was set to 90°C, if 90°C was reached, power turned off automatically. After microwave assisted extraction, all suspensions were mixed and centrifuged at 4600 r/m and 20°C for 30 min. The pellet was discarded and the supernatant was kept at 4°C until purification. To precipitate alginate, CaCl_2_ was added to a final concentration of 2 (% w/v) and the mixture was stored at 4 °C overnight. The mixture was centrifuged at 4600 r/m 20°C for 30 min and the pellet was discarded. The suspension was dialyzed in dialysis bags (Spectra/Por 3, MWCO: 3500 Da) for 48 h in Mili Q water with water changes every 12 h. To precipitate polymers, EtOH was added to a final concentration of (72% v/v) and precipitation occurred overnight at 4°C. Fucoidan extract was harvested by centrifugation at 4600 r/m and 20°C for 30 min. The pellet was freeze-dried (Christ, Gamma, 1–16, LSC) and the *Fucus* extract (EXT) was stored at −20°C.

### Determination of polymer monosaccharide composition

To analyse the monosaccharide composition of fucoidan, *Fucus* extract, samples (1 mg) were hydrolyzed with 450 μL (2.5 M) trifluoroacetic acid at 121°C for 2 h (Memmert, UF55) and dried with a N-EVAP 112 nitrogen evaporator (Organomation) at 50°C. Using the sample hydrolysis procedure, we determined the fucose content within the dry MacFarlane medium components (outlined below) including carbohydrate and peptide sources. Dried samples were re-suspended in 1 ml MilliQ water, vortexed and passed through a 0.45 μm syringe filter with a nylon membrane. L-fucose (Merck) was processed the same way for comparison.

Neutral monosaccharides were analysed on a Dionex ICS-6000 high-performance anion exchange chromatography coupled with a pulse amperometric detector (HPAEC-PAD) (all Thermo Scientific). The system was equipped with a Single Pass/Double Pass pump working as single pass, an AS-AP autosampler, a 10 μL injection loop and an ICS 6000 electrochemical detector operated with a gold electrode and an AgCl reference electrode. Neutral monosaccharides were separated at 25°C using a Dionex CarboPac PA-1 (2×250 mm) column attached to a Dionex CarboPac PA-1 guard column (2×50 mm) at a flow rate of 0.25 ml min^−1^. The mobile phase consisted of nitrogen degassed solvent A (MiliQ water), and 9% solvent B (200 mM NaOH) run in isocratic mode. The system was controlled by Chromeleon 7.2 SR4 (Thermo Scientific). Monomers were identified with external standards and all samples were analysed in triplicates.

### Identification of functional chemical moieties by Fourier transform infrared (FT-IR) spectroscopy

A NICOLET Summit infrared spectroscopy (Thermo Scientific) equipped with a diamond crystal ATR Everest probe and under control of Omnic Paradigm software was used to perform FT-IR spectroscopy analysis of dried fucoidan, *Fucus* extract and for comparison, fucose. Spectra were collected between 400 and 4000 cm^−1^ at a resolution of 2 cm^−1^ averaging 32 scans.

### CHNS element analysis

Carbon, hydrogen, nitrogen and sulfur (CHNS) composition of the extract was analysed using a VarioMacrocube (Elementar) calibrated on a sulfanilamide standard after drying the samples in foil at 100°C overnight. Samples were prepared by weighing 23, 22, 21.4, and 7 mg of dry mucin, fucose, fucoidan, and *Fucus* extract, respectively, into tin foil capsules, along with one pinch of vanadium pentoxide that was required to combust the sulfur.

### Preparation of fermentation media

Fecal slurries were cultivated in modified MacFarlane medium with extra buffer capacity (Bircher et al. 2017) containing complex carbohydrates and nitrogen sources to mimic substrates that are available in the large intestine. The medium composition (g L^−1^) was as follows in MilliQ water: 1.0 cellobiose, 1.0 xylan, 1.0 arabinogalactan, 0.5 inulin, 1.0 soluble starch, 3.0 amicase, 5.0 bacto tryptone, 1.5 meat extract, 4.5 yeast extract, 4.0 porcine mucin, 0.005 hemin, 0.4 bile salt, 3.0 KH_2_PO_4_, 9.0 NaHCO_3_, 0.05 MgSO_4_, 0.5 CaCl_2_ x2H_2_O, 1.0 MnCl_2_x4H_2_O, 0.025 FeSO_4_x7H_2_O, 0.5 ZnSO_2_x7H_2_O, 4.5 NaCl, 4.5 KCl. Tween 80 (1 ml) was added together with volatile fatty acids for a finale concentration of 33 mM acetate, 1 mM isobutyrate, 1 mM isovalerate, 1 mM valerate and 9 mM propionate. The medium was divided into four parts to be used as controls (CON), or with the addition of 0.4 (FUS0.4), 0.8 (FUS0.8), or 1.6 (FUS1.6) g L^−1^ fucose, 0.4 g L^−1^ fucoidan (FUC) or *Fucus* extract (EXT). The pH of the medium was adjusted to 7.1 with 3.0 M NaOH before boiling for 15 min for a final pH of around 6.5 after autoclaving. The medium was cooled and flushed with CO_2_ before adding 0.5 g L^−1^ cysteine-HCl and 0.1 ml L^−1^ vitamin solution. Media were dispensed in 20 ml serum flasks in aliquots of 10 ml (CON, FUS0.4–1.6, FUC) and 5 ml (EXT) with CO_2_ flow. Serum flasks were sealed with butyl rubber septums and aluminium caps before autoclaving at 121°C for 15 min.

### Donor recruitment and fecal sample processing

Fresh fecal samples were collected from healthy donors over a period of five months in Aarhus, Denmark. Anonymous sample collection and further processing is exempt from ethic approval according to the National Scientific Committee (National Videnskabsetisks Komite, NVK, Denmark). The donors were between 20–49 years and had regular eating patterns and bowel movements. Donors did not take any food supplements containing prebiotics or probiotics nor used any medication affecting the gut transit and digestion during the last three months preceding the sample donation. All donors provided written consent.

Samples were obtained over two sampling campaigns (experiment 1 and 2), and donors were not discouraged from donating in both experiments. In total, 17 fresh fecal samples were collected. Each fecal sample was immediately transferred to a sealed bag containing an anaerobic gas pack (BD) and was processed within 4 h of defecation. To prepare fecal slurries, all work was conducted in an anaerobic bench (Baker Ruskinn) in order to keep a strict anaerobic environment. Approximately 1 g of fresh fecal sample was added to a tube containing four sterile glass beads for breaking down the structure. Peptone water was added to obtain a 10% (m/v) solution. The samples were vortexed for 5 min in order to create a homogenous mixture and were left for 5 min for the larger particles to sediment before inoculation. Aliquots were collected from fecal samples and stored at −20°C for DNA isolation.

### *In vitro* batch fermentations

*In vitro* in batch fermentations were conducted with freshly prepared fecal slurries as inoculum to investigate the effect of fucoidan as a prebiotic. Media were inoculated with 1% fecal slurry. CON, FUS0.4 and FUS0.8 fermentations were run in triplicates, EXT was run in duplicates in experiment 1. In experiment 2, CON and FUS were always fermented in triplicates, while FUC and FUS1.6 were run in duplicates for three donors. There was no FUC incubation for donor sample 17.

Flasks were placed in a shaking incubator at 140 r/m and 37°C. Samples were collected after 0, 24, and 48 h of fermentation for further analyses. For H_2_S analysis, fresh fermentation broth was used as outlined below. Additional broth was collected, supernatant and biomass were separated by centrifugation and stored at −20°C for analysis of fermentation metabolites and DNA isolation, respectively. The pH was determined after 48 h of fermentation in experiment 1 using a FiveEasy pH meter F20 (Mettler Toledo).

### DNA extraction from feces and *in vitro* batch fermentations

DNA were both extracted from fecal samples and pellets collected from fermented FUS1.6 samples after 48 h (1 ml) with the FastDNA Spin Kit for soil (MP Biomedicals), which includes a bead beating step, and was eluted in 50 μL elution buffer. The extracted DNA was diluted 10-fold for all further assays.

### Microbiota profiling with 16S rRNA gene sequencing and data analysis

For library preparation, a two-step PCR approach was used according to Illumina's 16S Metagenomic Sequencing Library Preparation guide. Briefly, the V3-V4 hypervariable region of the 16S rRNA gene was amplified using Bac341F and Bac805R with adapters (Table S1) and a master mix containing 12.5 μL 2xKAPA HiFi HotStart readyMix (Roche) 0.5 μl forward and reverse primer, 1 μl DNA and 10.5 μl nuclease free water. PCR conditions were 25 cycles of denaturation at 95°C for 30 s, annealing at 55°C for 30 s and extension at 72°C for 30 s followed by 72°C for 5 min. The second PCR employed primers with barcode and amplification for 8 cycles. Samples were purified using Ampure XP beads (Beckman Coulter, Denmark) before sequencing. A pooled library comprising the amplicons of all samples was used for sequencing on a MiSeq sequencer (Illumina) at the Section of Microbiology at Aarhus University according to standard Illumina protocols. All samples including a negative control starting from a mock DNA isolation procedure and 61 samples from feces and fermentations were analysed in the same run and served as input for further bioinformatics processing.

Primer sequences (Table S1) were removed using cut adapt (v4.2; -O 12 –discard-untrimmed -g CCTACGGGNGGCWGCAG -G GACTACHVGGGTATCTAATCC –pair-adapters –minimum-length 75) (Martin 2011) and only inserts that contained both primers and were at least 75 bases were kept for downstream analysis. Reads were quality filtered using the filterAndTrim function of the dada2 package (maxEE = 2, truncQ = 3, minLen = 150, trimRight = 40, trimLeft = 40). The learnErrors and dada functions (Callahan et al., 2016) were used to calculate sample inference using pool = pseudo as parameter. Reads were merged using the mergePairs function and bimeras were removed with removeBimeraDenovo (method = pooled). Remaining Amplicon Sequence Variants (ASV) were taxonomically annotated using the IDTAXA classifier (Murali et al. 2018) in combination with the Silva v138 database (Quast et al. 2013). The median number of reads per processed sample was 26.904 (range 16.228–33.789 reads), the negative control yielded 563 reads. One fermentation sample was removed from further analysis as it failed sequencing.

### Quantification of specific functional bacterial groups

We used quantitative PCR (qPCR) to analyse counts of total bacteria and the abundance of specific microbial groups. To quantify total bacteria, the 16S rRNA gene was used as target (Table S1). For sulfate-reducing *Desulfovibrionaceae*, both the 16S rRNA gene and the gene *dsrA* encoding subunit A of the dissimilatory sulfate reductase were employed (Table S1). Abundance of *A. hallii* was determined using *pduC*, the gene encoding the major subunit of the propanediol/diol dehydratase as described (Ramirez-Garcia et al. 2021) (Table S1). A CFX Connect Real-Time PCR System (Bio-Rad) was used. The qPCR master mix contained 5 μL iTag Universal 2x SYBR Green Supermix (Bio-Rad), 1 μL forward and reverse primer (Table S1), 2 μL nuclease free water and 1 μL of diluted DNA in a total volume of 10 μL in 96-well (low profile clear/clear) PCR plate, sealed with Microseal ’B’ Sealing film (both Bio-Rad).

Each sample was run in duplicates, each run included a standard and a negative control (nuclease free water). The standard curves were made with linearized plasmids or purified PCR products. Reactions were run with the following temperature profiles: one cycle of hot-start activation at 95°C (3 min), denaturation at 95°C for 10 s, annealing at 60°C for 30 s for 40 cycles followed by melting curve analysis. Absolute cell abundance was calculated based on standard curves, correction factors were used to account for multiple 16S rRNA gene copies in gut microbes (Stoddard et al. 2015, Table S1).

### Metabolite analysis by high performance liquid chromatography coupled to a refractive index detector (HPLC-RI)

The concentrations of main SCFAs acetate, propionate and butyrate along with fucose, and lactate in feces and fermentation samples were determined by HPLC-RI using a 1260 Infinity II LC System with RID (all Agilent). Fermentation metabolites were separated using a Hi-Plex H column (300×7.7 mm) attached to a guard (50×7.7 mm) column.

To extract fermentation metabolites from feces, 200-300 mg material was mixed with 5 mM H_2_SO4, vortex and centrifuged 10 000 r/m, 10 min. Supernatants from batch fermentations were collected by centrifugation at 10 000 r/m for 4 min. All samples were filtered through a 0.45 μm nylon membrane filter before analysis. The samples (10 μL injection volume) were eluted with 5 mM H_2_SO_4_ at a flow rate of 0.6 ml min^−1^ at 40°C. Fermentation metabolites were quantified using external standards.

### Photometric determination of H_2_S in water

The H_2_S concentration was measured in fermentation samples collected at 0 and 48 h. The assay was adapted from Cline (1969) that is based on the reaction of H_2_S with N, N-dimethyl-1,4-phenylendiamin that produces methylene blue om the presence of iron (III) (Fe3+). The absorbance of the complex at 670 nm is proportional to the H_2_S concentration. Briefly, fermentation samples (0.5 ml) were recovered from closed serum flasks and were immediately dispersed in 0.5 ml 5% (w/v) zinc acetate to entrap the H_2_S. Samples were vortexed and stored at −20°C until further processing. After thawing, 20 μL was mixed with 980 μL MilliQ water. Diamine reagent (80 μL) was added, samples were shaken and placed in the dark at room temperature for 30 min. After, 300 μL were transferred to a 96 well microtiter plate together with a standard curve prepared using zinc sulfide, and absorbance was measured in a FLUOstar Omega plate reader (BMG Labtech) at 670 nm.

### Statistics

For α-diversity analysis, richness and evenness were calculated with the vegan package (Oksanen et al. 2023). Samples were normalized with rarefaction based on the minimal sum of all reads in the sample (n=16.228). For β-diversity analysis, coordination of samples were conducted based on Euclidean distance using non-metric multidimensional scaling (NMDS), which is for datasets with multiple dimensions including distance. The significance of coordination by different treatments was tested with Permutational Multivariate Analysis of Variance (PERMANOVA).

The relationship of microbial composition in fecal samples and of major fermentation metabolites formed in fermentations was determined using Factor Analysis for Mixed Data (FAMD) using the R packages FactoMineR and Factoextra.

Statistical analysis of fermentation metabolites and microbial abundance data was performed using ANOVA and paired t-test implemented in PAST (Hammer et al. 2001) and SigmaPlot V15 (Alfasoft).

## Results and discussion

### *Fucus* extract contained laminarin and fucoidan

While there have been reports that fecal microbiota, or selected gut microbes can, at least selectively, utilize algal polysaccharides (Cherry et al. 2019), there has been no systematic development and testing to position fucoidan as prebiotic, and no comparison to the major monomer L-fucose, possibly partly due to low commercial availability. This led us to investigate isolation of fucoidan in this study. The dry matter of *F. vesiculosus* used for the extraction was 19.0%. Employing a microwave assisted extraction procedure, 142 mg of extract was obtained with a yield of 0.2% (w/w% dry weight). The fucose content in the extract was analysed with HPAEC-PAD, together with the commercial fucoidan and fucose. Fucose contained mainly fucose (92.9±0.7%) whereas the commercial fucoidan had a fucose content of 78.6±2.2% and contained between 2.4% and 5.7% of galactose, mannose, rhamnose, glucose and xylose (Table S2). The *Fucus* extract contained 11.1±2.9% fucose and mainly glucose (70.9±3.6%) (Table S2).

The seaweed extraction procedure used in this study was adapted from Fletcher et al. (2017) and modified to the use of microwave assisted extraction according Ptak et al. (2019) albeit with modifications. Instead of 0.01 M H_2_SO_4_, 6% (w/v), at 120°C for 30 min, 0.1 M HCl, 3.3% (w/v) at 90°C for 15 min was applied together with lower temperature during microwave assisted extraction, which can be one of the reason for the low fucoidan yield obtained. Furthermore Ptak et al. (2019) neutralized pH to 5–7 after microwave assisted extraction and removed laminaran with 40% (v/v) EtOH before the precipitation, which was not done in this study. The high proportion of glucose in seaweed extract indicated that the *Fucus* extract was rich in laminarin and contained about 10% fucoidan.

### Sulfur was present in fucoidan and the *Fucus* extract

To examine the element composition with a focus on sulfur content, CNHS element analysis was conducted of fucose, commercial fucoidan, *Fucus* extract, and mucin for comparison (Table S3). Fucoidan contained 9.39% S, *Fucus* extract 0.29%, mucin 0.57% and fucose 0.04%. The minimal sulfur content of the medium, which could be derived from sulfate, was calculated based on the added levels of mucin, fucoidan and *Fucus* extract as at least 22.7–22.8 mg L^−1^ (or 1.4 mM) for CON and FUS0.4-FUS1.6, 60.8 mg L^−1^ (or 3.8 mM) for FUC and 23.8 mg L^−1^ (or 1.5 mM) for EXT.

To further investigate the presence of sulfur and fucose in the *Fucus* extract and the nature of its chemical bond, we performed FT-IR spectroscopy (Fig. S2A, B). With reference to the fucoidan spectrum, we attributed the peak at 1224 cm^−1^ and its shoulder at 1250 cm^−1^ to S-O stretching of sulfate ester groups, and the peaks at 893 cm^−1^ and 830 cm^−1^ to C-O-S stretching (Fig. S2B) (Ptak et al. 2021, Almeida-Lima et al. 2010). *Fucus* extract showed a clear peak at 1250 cm^−1^. The peaks associated with C-O-S bonds were less evident. While the peak at 884 cm^−1^ can indicate the presence of C-O-S bonds typical of fucoidan (shifted with respect to 893 cm^−1^), it was probably associated to the anomeric structure of glucose (880–889 cm^−1^) (Rajauria et al. 2021). This latter interpretation, together with the presence of peaks near 1420 cm^−1^ and 1620 cm^−1^ is compatible with the presence of laminarin in the sample (Synytsya and Novak 2014, Rajauria et al. 2021).

Similarly, the C-O-S peak at 830 cm^−1^ was not or only weakly present. Given the complexity of the peaks in the fingerprint region observed by FT-IR spectroscopy, and the overlapping of bands that can be attributed to different bonds (e.g. peaks at 1129 cm^−1^ and 893–876 cm^−1^), it was not possible to undoubtedly substantiate the sulfur bonds in the *Fucus* extract. In any case, FT-IR analysis provided a strong indication that S-O (and C-O-S bonds) were present in fucoidan and to a lesser extent in *Fucus* extract in line with the findings derived from elemental analysis.

For fucose, the peak at 876 cm^−1^ was attributed to the unique characteristic vibrational band of OH deformation (Fig. S2B) (Kossack et al. 2013). Other bands characteristic of OH stretching were visible at 3400 cm^−1^, 3370 cm^−1^, 3320 cm^−1^, and 3240 cm^−1^. The bands at 1170 cm^−1^ and 964 cm^−1^ that were attributed to the CH_3_ stretching, and the band at 1129 cm^−1^ attributed to C-O-S or C-O-C stretching and deformation, were common to both fucose and fucoidan structures (Fig. S2A) (Kossack et al. 2013). The peak of OH deformation that is characteristic of fucose, was not present or shifted in the spectrum of the *Fucus* extract, which can be indicative of either the absence or a low concentration of fucose in the sample.

### Addition of fucoidan and *Fucus* extract had minor overall impact on SCFA profiles

To determine the fermentability of fucoidan and *Fucus* extract, we conducted *in vitro* batch fermentations using taxonomically diverse human fecal microbiota (Supplementary Results, Table S4, Fig. S3) as inoculum. In experiment 1, batch fermentations were conducted with fecal microbiota of nine donor samples (D1-D9) and were incubated for 48 h in MacFarlane containing fucoidan (FUC) or *Fucus* extract (EXT), while in experiment 2 donor samples D10-D16 were tested with FUC medium. Controls (CON) were without additional supplementation and did not contain detectable levels of free fucose. We estimate the fucose content of Mac Farlane medium components at 0.05±0.02%. The production of SCFA was measured after 24 and 48 h of fermentation by HPLC-RI. The pH was determined after 48 h for CON and FUS0.4: 6.3±0.1, FUC: 6.4±0.1, and EXT 6.2±0.2 (experiment 1).

**Figure 3.**
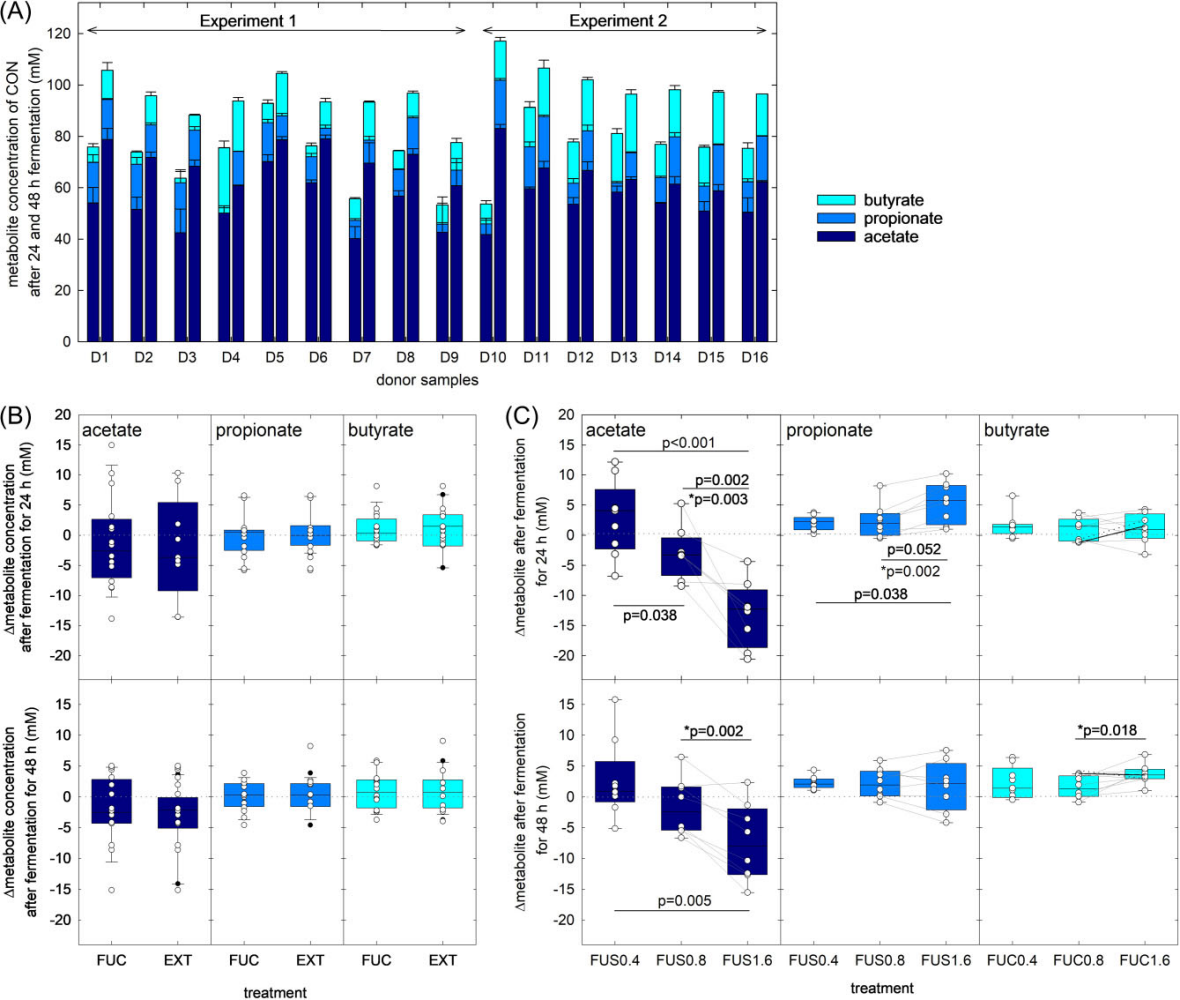
SCFA formation in experiment 1 and 2. Major SCFA acetate, propionate and butyrate formed in batch fermentations of donor samples D1-D9 (experiment 1) and D10-D17 (experiment 2) were determined using HPLC-RI. Fecal slurries were incubated in basic MacFarlane medium in the presence of 0.4 g L^−1^ (FUS0.4), 0.8 g L^−1^ (FUS0.8) or 1.6 g L^−1^ (FUS1.6) fucose or 0.4 g L^−1^ fucoidan (FUC) or *Fucus* extract (EXT) and compared to controls without supplementation (CON). Samples were fermented in triplicates unless indicated in the methods otherwise. (A) Major SCFA acetate, propionate and butyrate formed in CON fermentations after 24 (left bar) and 48 h (right bar). (B) Differences in metabolite formation between treatment samples FUC (D1-D17) and EXT (D1-D9) compared to CON after 24 (upper panel) and 48 (lower panel) of fermentation. (C) Differences in metabolite formation between treatment samples FUS0.4 (D1-D9), FUS0.8 and FUS1.6 (both D10-D17) compared to CON after 24 (upper panel) and 48 (lower panel) of fermentation. Box plots show median, 25^th^ and 75^th^ percentiles, the whiskers indicate 5^th^ and 95^th^ percentiles. Dots represent means of the individual donor fermentation that were run in triplicates or duplicates as stated in the methods. Differences between all fucose treatments (C) were determined using One-Way Anova with Holm-Sidak All Pairwise Multiple Comparison Procedures; differences between FUS0.8 and FUS1.6 were also tested with paired t-test. Treatments that differ significantly (*P* < 0.05) or with a trend (0.5<*P* < 0.1) are indicated in the graph; *indicates the p-value derived from the paired t-test.

In CON fermentations, the levels of total SCFA (acetate, propionate and butyrate) varied between 53.7–92.9 mM at 24 h and increased to 77.6–117.7 mM at 48 h suggesting that the major fermentation activity occurred during the first 24 h (Fig. 3A). Acetate was the major SCFA (63.8–81.0% and 60.5–84.7% at 24 and 48 h, respectively), followed by propionate (6.2–31.1% and 4.4–22.7% at 24 and 48 h, respectively) and butyrate (2.5–23.7 and 6.5–23.9% at 24 and 48 h, respectively) (Fig. 3A).

The release of fucose from fucoidan depends on the presence and activity of fucanases. Fucanases of glycosyl hydrolase (GH) families GH107 and GH168 cleave fucose from fucoidan polymers but were only described in environmental microbial isolates (Schultz-Johanson et al. 2018, Shen et al. 2020) and not in fecal microbiota. In contrast, GH29 and GH95 fucosidases that hydrolyse shorter fuco-oligosaccharides or terminal fucose, are frequently harboured by gut microbes (Wu et al. 2023). As the addition of fucoidan did not change median levels or proportions of propionate compared to controls in experiments 1 and 2 (Fig. 3B), our results suggest that fucose was not released from fucoidan at levels that would lead to detectable and consistent shifts of the SCFA profiles during the 48 h of incubation, which is in agreement with the generally low usability of fucoidan as prebiotic that was observed (Gotteland et al. 2020).

In a previous *in vitro* study, laminarin was completely utilized during fermentation when supplied as sole carbohydrate source (Devillé et al. 2007). While our analyses did not allow to determine the fate of the supplied polymers, lower median levels of Δacetate_treatment-control_ (FUC: -1.7 and -2.8 mM at 24 and 48 h, respectively, EXT: -3.7 and -2.2 mM at 24 and 48 h, respectively) indicate a rather fermentation inhibitory than fermentation supportive effect by the addition of fucoidan (FUC) or *Fucus* extract (EXT) (Fig. 3B).

### Fucose addition increased propionate levels

In both experiment 1 and 2 we also performed *in vitro* incubations that were supplied with fucose. In experiment 1, the supplied fucose (FUS0.4, 2.8 mM) was depleted after 24 h and the fermentation intermediate 1,2-PD was not detected in any sample while the fermentation intermediate lactate was detected in three samples only (3.3–15.2 mM). Complete fucose utilization and the lack of detection of 1,2-PD suggested that all donor microbiota were capable of fucose based cross-feeding.

With FUS0.4, total SCFA increased in 7/9 samples (ΔSCFA_FUS0.4-CON_ median 7.8 mM, Quartile (Q)1;3 -0.5;12.9 mM, Table S2) while the proportion of acetate was lower compared to CON (Δ%acetate_FUS0.4-CON_ median: -2.5, Q1;3: -2.7;-1,3%). Gut microbes produce propionate from the deoxyhexose fucose or from the fermentation intermediate 1,2-PD (Reichardt et al. 2014). Accordingly, the addition of fucose (FUS0.4) led to higher propionate levels (0.2–3.7 mM at 24 h, and 1.1–4.3 mM at 48 h) compared to CON (Fig. 3B), and to a higher proportion of propionate (Δ%propionate_FUS0.4-CON_ 24 h: median 2.1%, Q1;3 0.9;2.6%; 48 h: median: 1.7%, Q1;3: -0.5;2.5%). For FUS0.4, the level of fucose supplementation was nearly equimolar to the increase in propionate levels compared to controls similar as observed in co-culture studies of fucose-utilizing and 1,2-PD-producing *Bifidobacterium longum* subsp. *infantis* and the 1,2-PD utilizing *A. hallii* (Schwab et al. 2017). These results put forward that the addition of fucose enhanced propionate formation via the 1,2-PD pathway especially during the first 24 h of fermentation without (negatively) impacting propionate formation through the succinate or acrylate pathways.

As we observed in experiment 1 that the addition of fucose increased the formation of propionate in comparison to CON, we compared two higher concentrations of fucose (FUS0.8, 4.2 mM, and FUS1.6, 9.9 mM) to CON in experiment 2. Again, the supplied fucose was depleted after 24 h, and 1,2-PD was not detected in any sample; lactate was recovered from 9 samples (1.3–12.3 mM) after 24 h with no clear pattern related to treatment. While the addition of 0.8 g L^−1^ fucose did not lead to the expected overall higher propionate levels compared to FUS0.4, Δpropionate_FUS0.8/FUS1.6-CON_ levels were significantly (*P* < 0.05) higher in FUS1.6 compared to FUS0.4, and to FUS0.8 if paired t-test was used (Fig. 3C).

Median Δacetate levels of FUS0.8 were lower than CON at 24 h (Δacetate_FUS0.8 -CON_ median -3.3 mM, Q1;3 -6.7;-0.4 mM) and 48 h (Δacetate_FUS0.8-CON_ median -2.4 mM, Q1;3 -5.4;1.6 mM) and were significantly (*P* < 0.05, paired t-test) lower in FUS1.6 (Δacetate_FUS1.6-CON_ 24 h median: -12.3 mM, Q1;3 -18.6;-9.1 mM, 48 h median -8.0 mM, Q1;3 -12.6;-1.9 mM) compared to FUS0.8. After 48 h, butyrate levels of FUS0.8 (Δbutyrate_FUS1.6-CON_ median: 1.3 mM, Q1;3 0.1;3.4 mM) and FUS1.6 (Δbutyrate_FUS1.6-CON_ median: 3.5 mM, Q1;3 3.0;4.5 mM) were higher than CON, and butyrate of FUS1.6 was significantly (*P* < 0.05, paired t-test) higher than FUS0.8 (Fig. 3C).

Similar as reported by Ramirez et al. (2021), who supplemented intestinal microbiota with the fucose fermentation intermediate 1,2-PD, this study observed lower acetate levels and a higher proportion of butyrate in addition to propionate when fucose was added. Additionally, this effect was dose dependent with a significantly difference of acetate levels with higher levels of fucose (*P* < 0.05, Fig. 3C). As certain bacterial groups can produce butyrate from lactate and acetate (e.g. *A. hallii*), our observations point out that at the addition of fucose might impact on cross-feeding activities not only with 1,2-PD but also with lactate as intermediate, and that the effect is dose-dependent.

To identify if microbiota composition in the fecal sample (Supplementary Results, Fig. S3) related to fermentation metabolite formation, we conducted FAMD analysis. The first and second dimension explained 15.8 and 12.0% of the data (Fig. S4A, B). The variables ‘FUS0.4’ and ‘EXT’ were related to ‘*Coriobacteriaceae*’, ‘*Peptostreptococcaceae*’ and ‘*Bifidobacteriaceae’* with high contribution, while ‘FUS0.8’ and ‘FUS1.6’ related to ‘butyrate’ and ‘*Marinafilaceaea*’ indicating that donor sample microbiota composition contributed to fermentation metabolite profiles.

### Addition of fucose reduced H_2_S levels in a dose dependent manner

**Figure 4.**
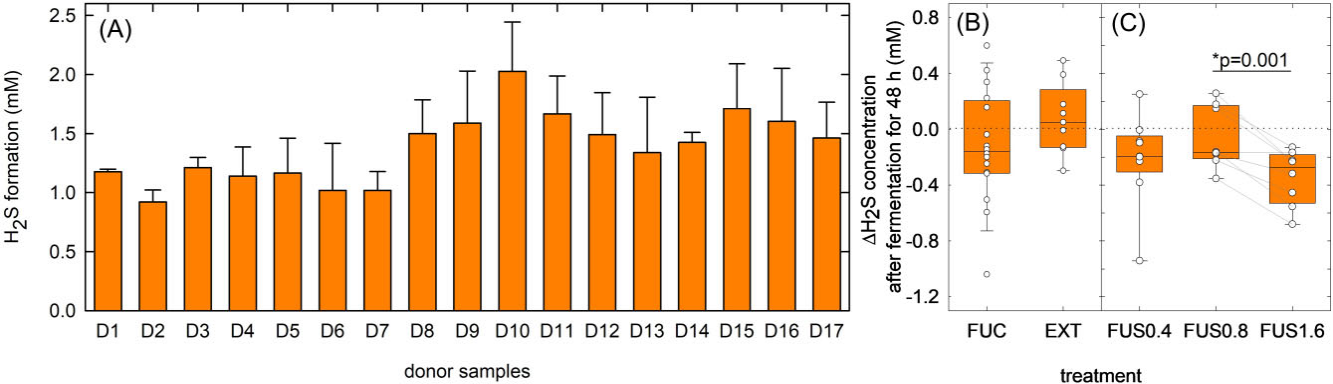
H_2_S formation in experiments 1 and 2. (A) H_2_S formation by donor samples D1-D17 were determined using a photometric assay. (B) Difference of ΔH_2_S levels after 48 h batch fermentations at 37°C of treatments FUC (from D1-D17) and EXT (from D1-D9) compared to controls. (C) Difference of ΔH_2_S levels after 48 h batch fermentations at 37°C of treatments of treatments FUS0.4 (D1-D9), FUS0.8 and FUS1.6 (both D10-D17). Samples were fermented in triplicates unless indicated otherwise in the methods, dots represent means of the triplicates. Lines connect samples from the same donor. Box plants show median, 25^th^ and 75^th^ percentiles, the whiskers indicate 5^th^ and 95^th^ percentiles. Differences between all fucose treatments (C) were determined using One-Way Anova with Holm-Sidak All Pairwise Multiple Comparison Procedures; differences between FUS0.8 and FUS1.6 were also tested with paired t-test. Treatments that differ significantly (*P* < 0.05) or with a trend (0.5<*P* < 0.1) are indicated in the graph; *indicates the p-value derived from the paired t-test.

As fucoidan rich in sulfate could serve as a source for the formation of H_2_S, the concentration of H_2_S was determined for all fermentations after 48 h of incubation with a photometric assay. In the colon, inorganic sulfate and sulfite, and sulfo amino acids such as cysteine act as sources for the production of H_2_S by sulfate reducers including dissimilatory sulfate reducers, or by utilizers of amino acids (Yao et al. 2018). Basic MacFarlane medium contained porcine mucin, tryptone and yeast extract which provide substrates for the production of H_2_S, and H_2_S formation was observed during fermentation in all samples (Fig. 4A). H_2_S concentrations of CON ranged from 0.92 to 2.03 mM (Fig. 4A) with no difference between CON, FUC (Δmedian -0.04 mM; Q1;3 -0.16;0.28 mM) and EXT (Δmedian 0.05 mM; Q1;3 -0.13;0.29 mM) (Fig. 4B). ΔH_2_S produced in the presence of FUS0.4 was 0.01–0.95 mM lower than CON (Δmedian -0.20 mM; Q1;3 -0.31;0.05 mM) and ΔH_2_S levels were lower for FUS1.6 (Δmedian -0.27 mM, Q1;3 -0.52;-0.17 mM) while ΔH_2_S_FUS1.6-CON_ was significantly lower (*P* < 0.05 mM) than ΔH_2_S_FUS0.8-CON_ (paired t-test; Fig. 4C).

As the addition of the heavily sulfated fucoidan or the laminarin-rich *Fucus* extract did not enhance H_2_S formation, our results indicate that bound SO_4_^2−^ was not a major contributor to H_2_S formation in batch cultures. Yao et al. (2018) observed that the addition of cysteine increased H_2_S formation by fecal microbiota more effectively compared to sulfate supplementation and proposed that a major part of intestinal H_2_S is formed from amino acids. The addition of fermentable FOS reduced H_2_S formation even when added together with cysteine as carbohydrates metabolism might be preferred over amino acids (Yao et al. 2018). Similarly, the addition of the fermentable deoxyhexose fucose might have contributed to the lower H_2_S formation observed in this study as a favorable substrate compared to amino acids.

### Fucose addition enhanced the abundance of selected bacterial groups linked to fucose utilization and cross-feeding

Next, we conducted 16S rRNA gene sequencing of biomass collected after 48 h batch fermentations of CON and FUS1.6 to determine how fucose addition impact microbial composition. We additionally quantified selected microbial groups (i.e. the hydrogen and propionate producing *A. hallii* and sulfate-reducing *Desulfovibrionaceae*) using qPCR to test whether the observed major differences in SCFA profiles and H_2_S formation were linked to differences in microbial abundance.

**Figure 5.**
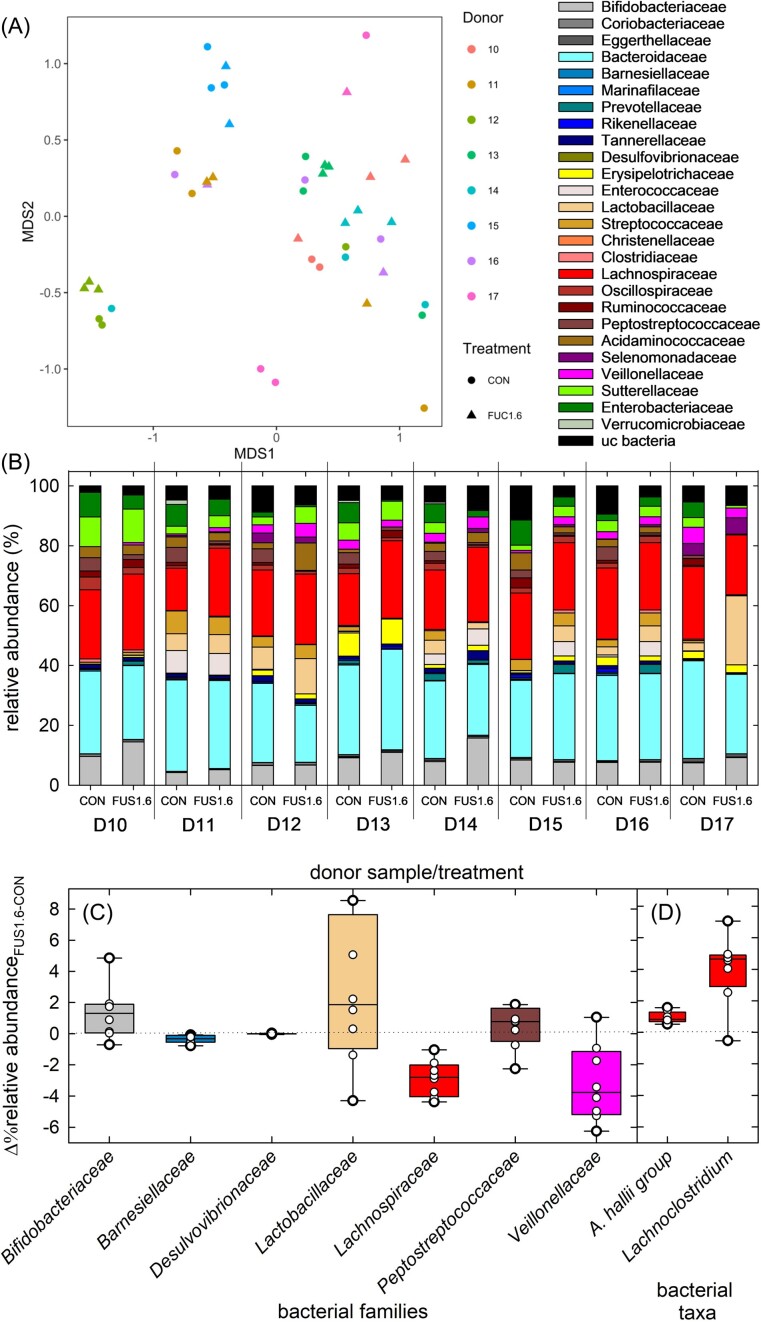
Differences in microbiota composition between CON and FUS1.6 (experiment 2). Microbiota composition was determined using 16S rRNA gene sequencing targeting the V3-V4 region. (A) Beta-diversity plots of CON and FUS1.6 of individual donor samples (D10-17, shown as 10–11) after 48 h fermentation. (B) Relative abundance of major families present in fermented samples CON and FUS1.6 was determined after 48 h incubations using 16S rRNA gene sequencing targeting the V3/V4 region. Relative abundance of selected bacterial families (C) and genera (D) that differed between CON and FUS 1.6after 48 h fermentation. Samples were fermented in triplicates unless indicated otherwise in the methods, differences were calculated from means. Box plants show median, 25^th^ and 75^th^ percentiles, the whiskers indicate 5^th^ and 95^th^ percentiles. Dots represent means of the individual donor fermentation that were run in triplicates or duplicates.

Based on α-diversity analysis, the median number of observed species (170, range 111–223) was significantly (*P* < 0.05, Kruskal–Wallis test with Dunn's posthoc test) lower for fermentation samples compared to feces (Table S4). Similarly, both Shannon and Simpson indices were lower in fermented samples than in feces (Table S4). When compared between treatments, the number of observed species was higher in CON samples than in FUS1.6 (Table S4). For β-diversity analysis, the significance of coordination by different donors and sampling time (t=0 h, fecal samples, and t=48 fermentations) were tested with permutational multivariate analysis of variance (PERMANOVA). The distance was significantly coordinated by time and donors (*P*=0.001) (Fig. S5) but not by treatment (Fig. 5A).

In CON fermentations, the most relative abundant bacterial families were *Bacteroidaceae* and *Lachnospiraceae* (Fig. 5B). Next, we calculated the differences in means of the independent fermentations for each donor to identify differences in relative abundance of CON and FUS1.6 (Fig. 5C). *Bifidobacteriaceae* (Δmedian 1.3%; Q1;3 0.1;2.6%), *Lactobacillaceae* (Δmedian 1.6%; Q1;3 -0.0;4.7%), *Lachnospiraceae* (Δmedian 1.9%; Q1;3 -0.1;5.9%) and *Veillonellaceae* (Δmedian 0.8%; Q1;3 0;1,2%) where higher in FUS1.6 than CON (Fig. 5C). The higher abundance of *Lachnospiraceae* in FUS1.6 was mostly due to the *A. hallii* group (Δmedian 0.8; Q25;75 0.7;1.1%) and *Lachnoclostridium* (Δmedian 4.6; Q25;75 3.7;4.8%) (Fig. 5D). When quantified with qPCR, median abundance of *A. hallii* (median 8.25 cells mL^−1^, Q1;3 8.13;8.35 cells mL^−1^) was significantly (p>0.05, Mann Whitney test) higher in FUS1.6 batch fermentations compared to CON (median 7.75 cells mL^−1^, Q1;3 7.63;7.90 cell mL^−1^).

Our analysis thus identified bacterial groups that have been previously linked to fucose utilization and cross-feeding: Formation of propionate and propanol from fucose has been shown for gut bacteria belonging to the family *Lachnospiraceae*, including *Roseburia* species (Scott et al. 2006) or *Lachnoclostridium* (Petit et al. 2013). *Bifidobacterium* spp. and *Lacticaseibacillus rhamnosus* are able to degrade fucose to 1,2-PD, or to 1,2-PD and lactate (Becerra et al. 2015, Bunesova et al. 2016), which can serve as metabolite in 1,2-PD cross-feeders such as *A. hallii, Blautia obeum, Ruminococcus gnavus, Flavonifractor plautii* and *Limosilactobacillus reuteri* (Engels et al. 2016, Schwab et al. 2017, Zhang et al. 2019) or lactate utilizers including again *A. hallii* and *Veillonella* spp. which produce butyrate or propionate, respectively (Fig. 1) (Ng and Hamilton 1971, Duncan et al. 2004). We previously identified *A. hallii* as a key taxon in the metabolism of 1,2-PD and glycerol, which are both catalyzed by the enzyme glycerol/diol dehydratase (Ramirez Garcia et al. 2021), and also observed an increase of abundance of the *A. hallii* group in this study. *A. hallii* can produce butyrate from lactate and acetate (Duncan et al. 2004), which might have contributed to the lower acetate levels in FUC1.6 samples.

### Fucose addition led to lower abundance of utilizers of sulfated amino acids

In addition, our 16S rRNA gene analysis revealed that the abundance of *Barnesiellaceae* (Δmedian -0.3%; Q1;3 -0;5,-0.1%), *Peptostreptococcaceae* (mostly *Intestinibacter bartlettii*, Δmedian -2.8%; Q1;3 -3.8;2.3%) and *Enterobacteriaceae* (mostly *E. coli/Shigella* group, Δmedian -3.8%; Q1;3 -5.0;-1.5%) was lower in FUS1.6 compared to CON (Fig. 5B). The abundance of *Desulfovibrionaceae* was quantified targeting the 16S rRNA gene (CON, median 7.30 cells mL^−1^, Q1;3 7.10;7.34 cells mL^−1^, FUS1.6, median 7.34 cells mL^−1^, Q1;3 7.21;7.47 cells mL^−1^) and *dsr* (CON, median 7.24 cells mL^−1^, Q1;3 7.04;7.41 cells mL^−1^, FUS1.6, median 7.11 cells mL^−1^, Q1;3 6.84;7.24 cells mL^−1^) and there was no difference in abundance between CON and FUS1.6 after 48 h of incubation.

Using an *in silico* approach, Braccia et al. (2021) reported that cysteine degraders are common within the human microbiota and more abundant than sulfate-reducing bacteria, which is in agreement with the low abundance of *Desulfovibrionaceae* observed in this study. *Enterobacteriaceae* represent a taxon that harbors a diversity of genes encoding enzymes involved in cysteine degradation and H_2_S production (Braccia et al. 2021). We observed a lower relative abundance of *Enterobacteriaceae* in FUS1.6 fermentations, which could be due to a competitive disadvantage of utilizers of cysteine or other sulfo amino acids in the presence of carbohydrates in agreement with Yao et al. (2018). *Veillonella* spp., whose relative abundance was higher in most FUC1.6 samples, can concurrently utilize lactate to form propionate, acetate and H_2_ (Distler and Krönke 1981) and metabolise cysteine to produce H_2_S (Washio et al. 2014). The extent of cysteine metabolism depends on growth state (resting cells versus cell extract), pH (more H_2_S formed at pH7 than pH5) and lactate levels (significant higher levels formed in the presence of 10 mM lactate) (Washio et al. 2014). The overall lower H_2_S levels in FUC1.6 samples indicate that the higher abundance of *Veillonella* was rather linked to lactate crossfeeding.

## Conclusion

The addition of fucose had a major impact on fermentation metabolite cross-feeding via both 1,2-PD and lactate and was also linked to alterations of microbial community composition. We show for the first time that addition of fucose reduced H_2_S formation possibly due to preferred utilization of the provided carbohydrate compared to amino acids. While it was previously shown that *A. hallii* can benefit within a microbial community from the utilization of the pathway intermediate 1,2PD, we report here that the species also benefits through cross-feeding during fucose metabolism, which might be relevant in the gut ecosystem.

The effects of fucose addition can be considered prebiotic with the observed increase of propionate and butyrate formation. However, this study highlights an important consideration in prebiotic research addressing whether human gut microbiota harbours the necessary enzymatic functionality to degrade specific polysaccharides such as fucoidan. As dietary fucose might be absorbed during gastrointestinal transit, and the corresponding fucoidan polymer seemed to be little utilized under the tested conditions, biotechnologically produced fuco-oligosaccharides could be the solution for precise fucose-based microbiome engineering strategies.

## Supplementary Material

fiad107_Supplemental_File

## Data Availability

16S rRNA gene libraries are available at the European Nucleotide Archive ENA under accession number PRJEB60530.
