# Supplementary material for: Fucose modifies short chain fatty acid and H2S formation through alterations of microbial cross-feeding activities
Source: FEMS Microbiol Ecol. 2023 Sep 30;99(10):fiad107. doi: 10.1093/femsec/fiad107 (PMC10561710; doi:10.1093/femsec/fiad107)
Supplement: fiad107_Supplemental_File [file fiad107_supplemental_file.docx]

Supplementary data for

**Fucose modifies short chain fatty acid and H_2_S formation through alterations of microbial cross-feeding activities**

Karina Høgsgaard^1^, Natalia P. Vidal^2,3^, Angeliki Marietou^1^, Oliver Gam Fiehn^1^, Qing Li^1^, Julia Bechtner^2^, Jacopo Catalano^4^, Mario M. Martinez^2^, Clarissa Schwab^1^

^1^ Functional Microbe Technology Group, Department of Biological and Chemical Engineering, Aarhus University, Gustav Wieds Vej 10, 8000 Aarhus, Denmark

^2^Center for Innovative Food (CiFOOD), Department of Food Science, Aarhus University, AgroFood Park 48, 9200 Aarhus N, Denmark

^3^Aarhus Institute of Advanced Studies, Aarhus University, Høegh-Guldbergs Gade 6B, 8000 Aarhus

^4^ Membrane Engineering Group, Department of Biological and Chemical Engineering, Aarhus University, Åbogade 40. 8200 Aarhus N, Denmark

**Supplementary Results**

**Microbiota composition of fecal donor samples**

To investigate the composition of the fecal microbiota of donor samples, we performed 16S rRNA gene sequencing of the V3-V4 region of the 16s rRNA gene. Samples contained between 96 and 358 species and had Chao1 index of 101-372 (**Suppl. Table S4**). *Bacillota* and *Bacteroidota* were the two most abundant bacteria phyla in all samples contributing a median of 67.5% (25 and 75% quartile (Q1;Q3) 57.5;73.9%) and 25.7% (Q1;3 18.1;28.7%), respectively. *Actinobacteria* (median 3.5%, Q1;3 2.6;5.2%), *Proteobacteria* (median 1.0%, Q1;3 0.3;1.3%), *Desulfobacterota* (median 0.1%, Q1;3 0.1;0.2%) and *Verrucomicrobia* (median 0.3%, Q1;3 0.01;1.2%) were present at low abundance.

On the family level, fecal microbiota was dominated by *Lachnospiraceae*, *Ruminococcaceae* (both *Bacillota*), and *Bacteroidaceae* (*Bacteroidota*) (**Suppl.** **Figure S3**). *Bifidobacteriaceae, Sutterellaceae, Desulfovibrionaceae* and *Verrucomicrobiaceae* were the most prominent families among the *Actinobacteria, Proteobacteria, Desulfobacterota* and *Verrucomicrobia,* respectively (**Suppl.** **Figure S3**)*.* Archaea of the *Methanobacteriaceae* were present in samples of four donors (0.2-1.2%). These observations indicate that a diverse fecal microbiota served as inoculum for batch fermentations.

**Supplementary Figures**

**Suppl. Figure S1. Flow diagram of fucoidan extraction procedure from *Fucus vesiculosus*.**


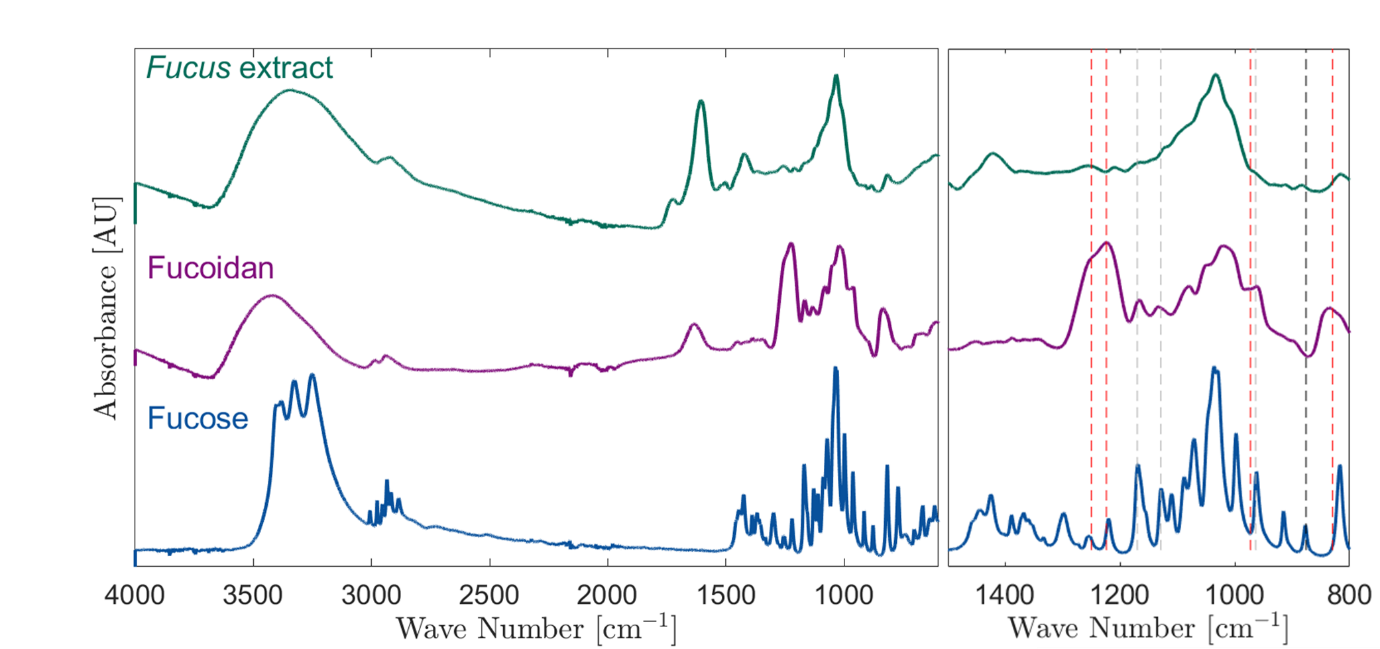


B

A

**Suppl. Figure S2**. **FT-IR analysis. (A)** Full FT-IR spectra (4000-400 cm^-1^) and **(B)** magnification of the fingerprint region (1500-800 cm^-1^) of *Fucus* extract, commercial *F. vesiculosus* fucoidan and L-fucose. Red reference lines are bands associated to the S-O stretching of sulfate ester groups (at 1224 cm^-1^ and 1250 cm^-1^ ) and C-O-S stretching (at 893 cm^-1^ and 830 cm^-1^) (Ptak et al., 2021; Almeida et al., 2010). The black reference line is associated with the OH deformation vibrational band (at 876 cm^-1^) characteristic of fucose (Kossack et al., 2013). Gray reference lines were attributed to the CH_3_ stretching and deformation (at 1170 cm^-1^ and 964 cm^-1^, respectively) and either to the C-O-S stretching or the C-O-C stretching (at 1129 cm^-1^) were present in both fucose and fucoidan structures.

**
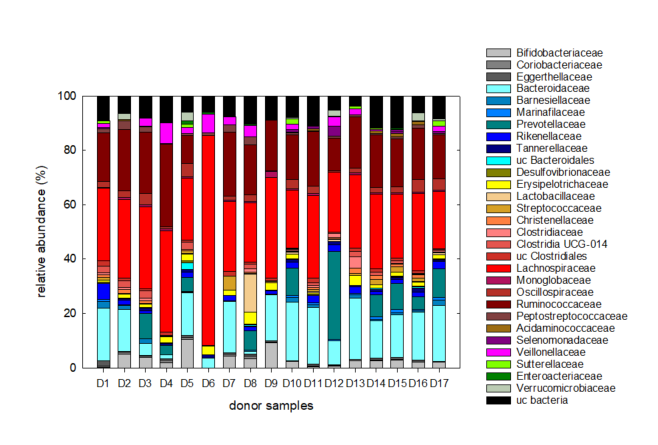
Suppl. Figure S3. Microbial composition in donor feces.** Relative abundance of major families present in feces of donors D1-D17 was determined using 16S rRNA gene sequencing targeting the V3/V4 region.

**A**

**
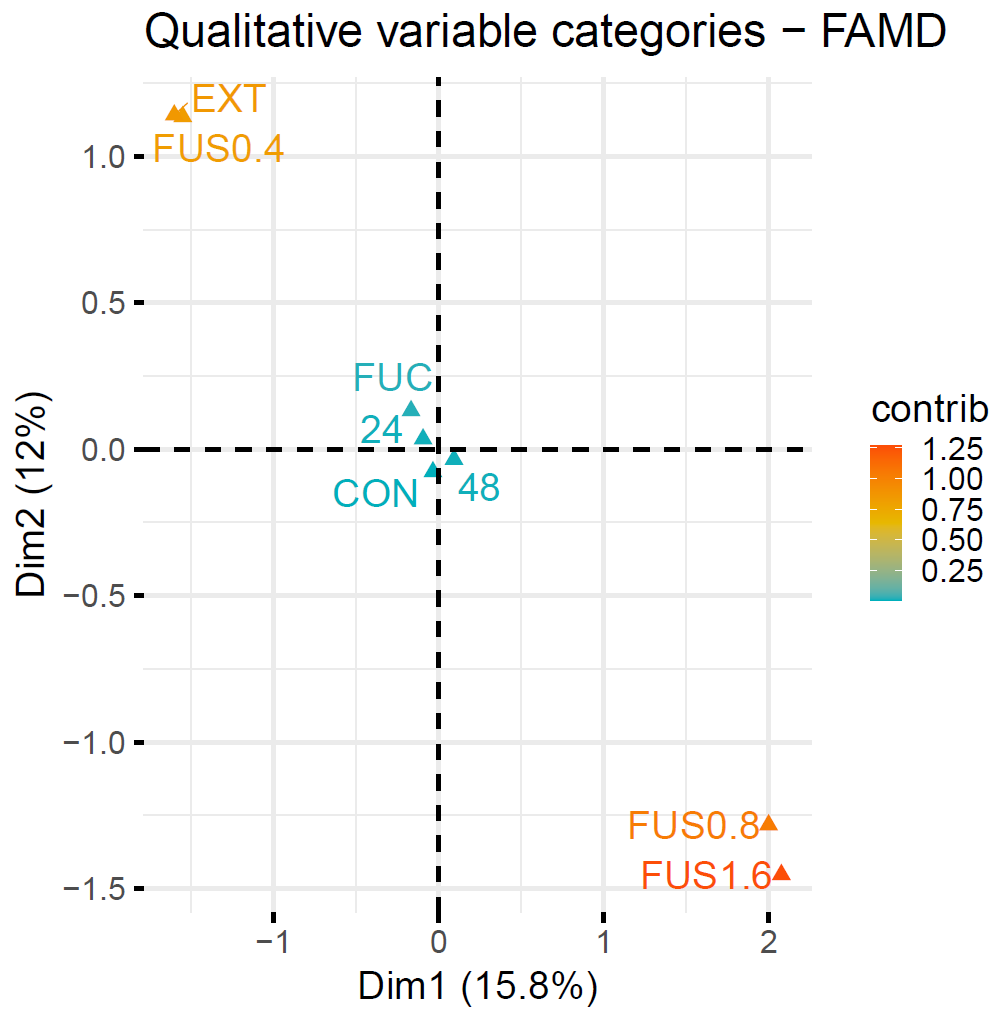
**

**B**

**
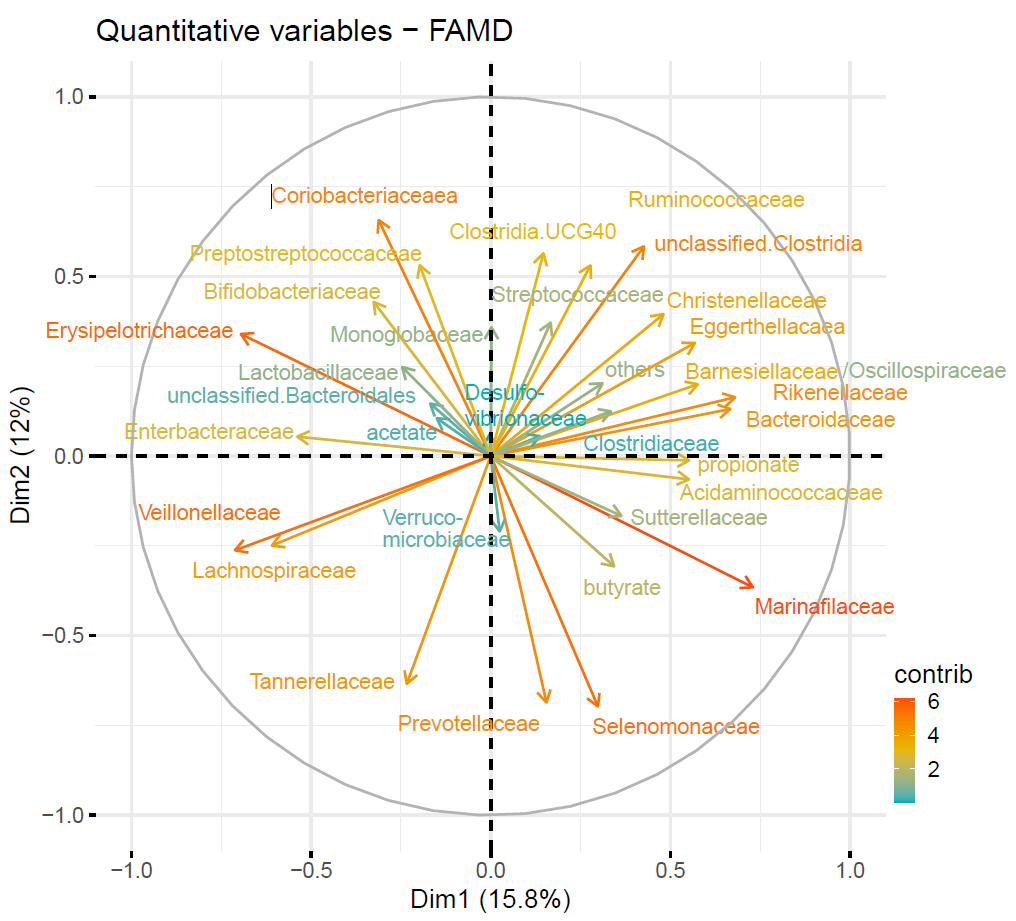
**

**Suppl. Figure S4. Relationship of fecal microbiota composition and fecal fermentation metabolites.** The relationship of relative abundance of microbial families present in fecal samples and of major fermentation metabolites formed after 24 and 48 h of incubation in control fermentations (CON) or MacFarlane medium supplement with fucoidan (FUC), *Fucus* extract (EXT), and fucose (FUS0.4, FUS0.8, and FUS1.6) was determined using Factor analysis for mixed data (FAMD). Treatment and time was used as categorical variables (A), while quantitative data was relative abundance and concentrations of SCFA (B).

**
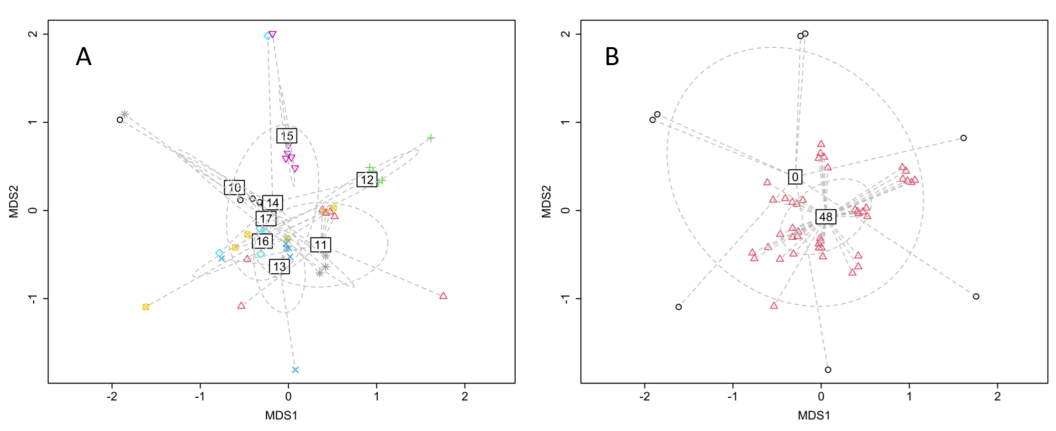
**

**Suppl. Figure S5. Beta diversity analysis.** Coordination of experiment 2 (CON and FUS2) that were grouped by donors (A) and time (B).

**Suppl. Table S1.** **Primer used for 16S rRNA gene sequencing and quantitative PCR (qPCR) of selected microbial groups.**

| **Purpose** | **Target group** | **Target gene** | **Primer name** | **Primer sequence (5’-3’)** | **Reference** |
| --- | --- | --- | --- | --- | --- |
| 16S rRNA gene sequencing | Bacteria+  archaea | 16S rRNA gene | Bac341F | CCT ACG GGN GGC WGC AG | - |
|  |  |  | Bac805R | GAC TAC HVG GGT ATC TAA TCC | - |
|  |  |  | Bac341F_adapter | TCG TCG GCA GCG TCA GAT GTG TAT AAG AGA CAG CCT ACG GGN GGC WGC AG | - |
|  |  |  | Bac805R_adapter | GTC TCG TGG GCT CGG AGA TGT GTA TAA GAG ACA GGA CTA CHV GGG TAT CTA ATC C | - |
| qPCR | Total bacteria | 16S rRNA gene* | Bac338F | ACTCCTACGGGAGGCAGCAG | - |
|  |  |  | Bac534R | ATTACCGCGGCTGCTGG | - |
|  | sulfate reducing *Desulfovibrionacae* | 16S rRNA gene* | DSV691F  DSV826R | CCGTAGATATCTGGAGGAACATCAG | Fite et al., 2004 |
|  |  |  |  | ACATCTAGCATCCATCGTTTACAGC- |  |
|  |  | *dsr* | Dsr1F | ACSCACTGGAAGCACGGCGG | Kondo et al., 2004 |
|  |  |  | Dsr1R | GTGGMRCCTGCAKRTTGG |  |
|  | *Anaerobutyricum*  *hallii* | *pduC* | pduF  pduR | CGTTATGCTCCATTTAATGCT  CCAAGGAGTATCATCACCATC | Ramirez-Garcia et al., 2021 |

*correction factor for multiple 16S rRNA genes (Stoddard et al., 2015), 5 for total bacteria, 4.5 for DSR

**Suppl. Table S2.** **Monosaccharide composition of fucoidan and *Fucus* extract.** Monosaccharide composition was determined after hydrolysis using ion-chromatography with pulsed amperometric detection. Fucose was included as control.

| **Source** | **Monosaccharide composition (mol%)** | | | | | |
| --- | --- | --- | --- | --- | --- | --- |
|  | Fucose | Galactose | Glucose | Mannose | Rhamnose | Xylose |
| Fucose | 92.9±0.7 | 2.2±0.6 | 1.1±0.2 | 0 | 3.8±0.4 | 0 |
| Fucoidan | 78.6±2.2 | 2.4±0.4 | 5.4±0.2 | 3.2±0.4 | 4.7±0.6 | 5.7±1.1 |
| *Fucus* extract | 11.1±2.9 | 5.1±0.3 | 70.9±3.6 | 5.4±2.5 | 0 | 7.5±6.6 |

**Suppl. Table S3.** **Element composition of fucoidan and seaweed extract.** Element composition was determined using CHNS analysis and was compared to porcine mucin and fucose.

| **Source** | **Element (%)** | | | |
| --- | --- | --- | --- | --- |
|  | N | C | H | S |
| Fucose | 0.07 | 43.76 | 8.09 | 0.04 |
| Fucoidan | 0.09 | 25.36 | 5.01 | 9.39 |
| *Fucus* extract | 1.14 | 38.79 | 3.65 | 0.29 |
| Mucin | 10.22 | 49.65 | 8.86 | 0.57 |

**Suppl. Table S4. Alpha diversity analysis of fecal and fermentation samples.** 16S rRNA gene composition was determined using 16S rRNA gene amplicon sequencing of the V3-V4 region. Differences between sample groups were determined using Kruskal-Wallis test with Mann-Whitney pairwise adhoc test. Different letters indicate significant (p<0.05) difference between groups.

| **Origin** | **Number of samples** | **Treat-**  **ment** | **Reads (median, range)** | **Richness (observed species, median, range)** | **Chao 1 (median, range)** | **Shannon index (median, range)** | **Simpson Index (median, range)** |
| --- | --- | --- | --- | --- | --- | --- | --- |
| Feces | 17 |  | 29.029, 22.167-33.789 | 266^A^, 96-361 | 274^A^,101-371 | 4.45^A^, 3.21-4.81 | 0.98^A^, 0.92-0.98 |
| Fermen-  tation | 43 | CON | 25.105, 16.228-31.013 | 173^B^, 157-223 | 182^B^, 130-235 | 3.84^B^, 3.49-4.10 | 0.96^B^, 0.94-0.97 |
|  |  | FUS3 | 26.457, 21.797-21.197 | 162^C^, 111-223 | 178^B^, 114-230 | 3.72^B^, 3.35-3.91 | 0.74^B^, 0.93-0.97 |

**Supplementary References**

Almeida-Lima J, Costa LS, Silva NB, et al. Evaluating the possible genotoxic, mutagenic and tumor cell proliferation-inhibition effects of a non-anticoagulant, but antithrombotic algal heterofucan. *J Appl Toxicol* 2010;*30*:708-15.

Christophersen CT, Morrison M, Conlon MA. Overestimation of the Abundance of Sulfate-Reducing Bacteria in Human Feces by Quantitative PCR Targeting the Desulfovibrio 16S rRNA Gene. *Appl Environ Microbiol* 2011;*77*:3544-6.

Fite A, Macfarlane GT, Cummings JH, et al. Identification and quantitation of mucosal and faecal Desulfovibrios using real time polymerase chain reaction. *Gut* 2004;*53*:523–9.

Kondo R, Nedwell DB, Purdy KJ, Silva SQ. Detection and enumeration of sulphate-reducing bacteria in estuarine sediments by competitive PCR. *Geomicrobiol J* 2004;*21*:145–57.

Kossack W, Adrjanowicz K, Tarnacka K, et al.½B N Glassy dynamics and physical aging in fucose saccharides as studied by infrared- and broadband dielectric spectroscopy.  *Phys Chem Chem Phys* 2013:*15*: 20641-20650.

Ptak SH, Sanchez L, Frette X, Kurouski D. Complementarity of Raman and infrared spectroscopy for rapid characterization of fucoidan extracts. *Plant Meth* 2021;*17*:130.

Stoddard SF, Smith BJ, Hein R, Roller BR, Schmidt TM. rrnDB: improved tools for interpreting rRNA gene abundance in bacteria and archaea and a new foundation for future development. *Nucl Acids Res* 2015;43:D593-8.
